# Recalibrating prognostic models to improve predictions of in‐hospital child mortality in resource‐limited settings

**DOI:** 10.1111/ppe.12948

**Published:** 2023-02-06

**Authors:** Morris Ogero, John Ndiritu, Rachel Sarguta, Timothy Tuti, Jalemba Aluvaala, Samuel Akech

**Affiliations:** ^1^ Kenya Medical Research Institute (KEMRI)‐Wellcome Trust Research Programme Nairobi Kenya; ^2^ School of Mathematics University of Nairobi Nairobi Kenya; ^3^ School of Medicine University of Nairobi Nairobi Kenya

**Keywords:** model recalibration, paediatric mortality, prediction

## Abstract

**Background:**

In an external validation study, model recalibration is suggested once there is evidence of poor model calibration but with acceptable discriminatory abilities. We identified four models, namely RISC‐Malawi (Respiratory Index of Severity in Children) developed in Malawi, and three other predictive models developed in Uganda by Lowlaavar et al. *(2016).* These prognostic models exhibited poor calibration performance in the recent external validation study, hence the need for recalibration.

**Objective:**

In this study, we aim to recalibrate these models using regression coefficients updating strategy and determine how much their performances improve.

**Methods:**

We used data collected by the Clinical Information Network from paediatric wards of 20 public county referral hospitals. Missing data were multiply imputed using chained equations. Model updating entailed adjustment of the model's calibration performance while the discriminatory ability remained unaltered. We used two strategies to adjust the model: intercept‐only and the logistic recalibration method.

**Results:**

Eligibility criteria for the RISC‐Malawi model were met in 50,669 patients, split into two sets: a model‐recalibrating set (*n* = 30,343) and a test set (*n* = 20,326). For the Lowlaavar models, 10,782 patients met the eligibility criteria, of whom 6175 were used to recalibrate the models and 4607 were used to test the performance of the adjusted model. The intercept of the recalibrated RISC‐Malawi model was 0.12 (95% CI 0.07, 0.17), while the slope of the same model was 1.08 (95% CI 1.03, 1.13). The performance of the recalibrated models on the test set suggested that no model met the threshold of a perfectly calibrated model, which includes a calibration slope of 1 and a calibration‐in‐the‐large/intercept of 0.

**Conclusions:**

Even after model adjustment, the calibration performances of the 4 models did not meet the recommended threshold for perfect calibration. This finding is suggestive of models over/underestimating the predicted risk of in‐hospital mortality, potentially harmful clinically. Therefore, researchers may consider other alternatives, such as ensemble techniques to combine these models into a meta‐model to improve out‐of‐sample predictive performance.

## 1 BACKGROUND

Prognostic models predict patients' risk of deterioration or poor outcomes and good models can inform clinical treatment or follow‐up plans.^1^ Developing new models without investigating the performance of existing models wastes potentially important historical data and research efforts.^2^ External validation of published prognostic models in populations/settings comparable to the model's derivation is recommended for establishing model transportability and generalisability.^3^, ^4^, ^5^

Most clinical prediction models may not perform well in external validation and end up being rejected because of poor predictive performance. This is partly because clinical environments continuously evolve in various ways, including shifts in clinical practice, even though clinical practice guidelines tend to standardise this.^6^ Other reasons include a change in patient management such as the use of aggressive treatment therapies, e.g., use of higher molecules of antibiotics as opposed to the first‐line, and the introduction of new vaccines, e.g., RTS,S/AS01 which is a world's first malaria vaccine.^7^ Such interventions may change the prevalence and clinical presentations of common childhood illnesses, and thus, a clinical prediction model developed before these interventions would perform poorly when validated in such settings. Variation in case‐mix, different time points of model development and validation, and dataset drift also contribute towards the deterioration of the model performance when applied in new samples hence a need for model recalibration to contextualise to the local settings.^8^

Model updating is suggested once there is evidence of poor model calibration but acceptable discriminatory abilities in an external validation study.^9^, ^10^ In the recent external validation study, we identified four prognostic models whose calibration estimates suggested an underestimation of in‐hospital paediatric mortality risk. These models included the Respiratory Index of Severity in Children (RISC‐Malawi) by Hooli et al. (2016)^11^ and three other models developed by Lowlaavar et al. (2016).^12^

In this study, we aim to recalibrate these models using regression coefficients updating strategy and determine how much their performances improve.

## 2 METHODS

### 2.1 Models' calibration metrics

The threshold for a perfectly calibrated score is a model with a calibration slope of 1 and calibration intercept (calibration‐in‐the large) of 0 or an identity line of 45° in the calibration plot indicating limited chances of over/underestimating the risk of bad outcomes when used in clinical practice. Although it is not clear how close these metrics should be to the set thresholds for the model to be acceptable, there is consensus from the literature that a model has good calibration if the intercept is close to 0 and the slope is close to 1.^13^ For instance, a model slope of 0.95 was termed “good calibration” by Philips et al.*,* ^14^ and Nakhjavan et al.^15^ termed a model with a slope of 0.97 and an intercept of 0.006 “proper calibration”.

### 2.2 Details of the models to be recalibrated

The RISC‐Malawi^11^ model and 3 models by Lowlaavar et al. (2016)^12^ were identified in an earlier review^3^, ^4^ highlighting models predicting in‐hospital paediatric mortality. RISC‐Malawi is a Respiratory Index of Severity in Children (RISC) developed using prospectively collected clinical data from a cohort of 14,665 hospitalised children aged 2–59 months with pneumonia in Malawi between 2011 and 2014. The three models by Lowlaavar et al. (2016)^12^ utilised a two‐site prospective observational study in Uganda that enrolled 1307 children between 6 months and 5 years admitted with a proven or suspected infection. A recent external validation study of these models suggested that while they had fair discriminatory ability (c‐statistics ranging from 0.70 to 0.79),^16^, ^17^, ^18^ they were poorly calibrated as judged from their calibration slopes and intercepts as shown in Figure S1.

The Kenya Medical Research Institute's Scientific and Ethical Review Committee approved the Clinical Information Network (CIN) project (#3459), whose data are used in the current study of recalibrating models.

### 2.3 Sources of data

To recalibrate the identified models, we used data collected by CIN, which comprises 20 public county referral hospitals in Kenya, and had 212,654 patients admitted between January 2014 and December 2021. In this network, patient details are systematically documented by duty clinicians and nurses who provide care in the hospitals using a standardised medical record known as the Paediatric Admission Record (PAR),^19^ that has been adopted for use by hospitals participating in CIN. Upon discharge or the death of a patient, a trained clerk abstracts data from the PAR and other medical notes into a customised data capture tool designed using a non‐proprietary Research Electronic Data Capture (REDCap) platform.^20^

### 2.4 Availability of model predictors in the recalibration cohort

For the RISC‐Malawi model, all predictors were available across all 20 hospitals contributing to the model's updating dataset except for the predictor called *unconsciousness*. We recoded this predictor based on the disability scale of AVPU (Alert, Verbal response, response to Pain, Unresponsive) such that a patient was assumed to be unconscious if the clinician‐rated them as either “P” (only responding to pain) or “U” (unresponsive). AVPU is known for the assessment of the patient's brain function and is therefore used for the determination of the level of consciousness.^21^ For the Lowlaavar models, all predictors were available in all hospitals except for the *Blantyre Coma Score,* which was available in only six hospitals for patients admitted as from September 2019.

The outcome to be predicted by the models was all‐cause in‐hospital paediatric mortality and was documented in each patient.

### 2.5 Eligibility criteria for model recalibration cohort

To determine appropriate patients to be included in the cohort of model recalibration, we applied the same eligibility criteria as were used in the original model derivation studies.^11^, ^12^ In summary, for the RISC‐Malawi model, we included children aged 2–59 months with an admission diagnosis of pneumonia defined as either cough or difficult breathing and any of the danger signs, namely central cyanosis, grunting, chest wall indrawing, stridor, inability to drink or breastfeed, convulsing, or not being alert based on the disability scale of the AVPU scale. For the Lowlaavar models, we included children aged 6–60 months admitted with any confirmed or suspected infectious diseases. To achieve these eligibility criteria, we filtered out all patients with non‐communicable diseases. In each of the two model recalibration cohorts, we excluded children admitted for surgery or with burns, trauma, road traffic accidents, poisoning such as organophosphate ingestion, and those patients admitted during the healthcare workers' strike.

**FIGURE 1:**
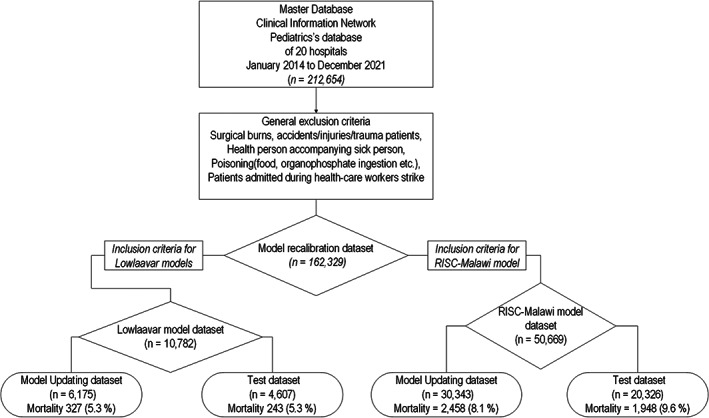
Populations used to update and test RISC‐Malawi model and 3 models by Lowlaavar et al 2016.

To estimate models' temporal transportability after recalibration, we split the data meeting the eligibility criteria into a model updating set (for recalibrating the model) and test set (for assessing model performance after updating) based on the time of patient admission.^22^ For the RISC‐Malawi model, 50,669 patients met the eligibility criteria; the updating set included 30,343 patients admitted across all 20 hospitals from January 2014 through December 2018, while its test dataset included 20,326 patients admitted in the same hospitals from January 2019 through December 2021. For Lowlaavar models, there were 10,782 patients meeting eligibility criteria. In all, 6175 of these patients admitted from September 2019 through December 2020 were used to update the models, while those admitted to the same hospitals from January 2021 through December 2021 (n = 4607) were included in the test set as shown in Figure 1.

### 2.6 Sample size for model recalibration

Following approaches described by Riley et al.^23^, ^24^ taking into account the c‐statistics of the original models, the number of parameters in the original model, and the prevalence of the outcome (in‐hospital mortality) in the derivation cohort, we computed the sample sizes required to recalibrate each of the four models assuming an acceptable difference of 0.05 between the apparent and adjusted *R‐squared* of the original model. Minimum sample sizes required for each of the 4 models are provided in the Table S1. For example, while sample size calculation approaches required a minimum sample size of 1619 for the RISC‐Malawi model, our model updating and test datasets exceeded this. In addition, with mortalities of >1000 in RISC‐Malawi model datasets and >200 in Lowlaavar model datasets, the events‐per‐variable ratio exceeded the recommended ratio of 20.^23^, ^25^

### 2.7 Assessment of missing data in the model recalibration cohort

In model recalibration computations, all prognostic factors are expected to have data for each patient in a cohort, otherwise records with incomplete data are deleted from the analysis resulting in “complete case analysis”, which could lead to a loss of statistical power and potentially yield biased estimates.^26^ Missing data assessment suggested that 68.3% and 5.2% of the patients' records for updating the RISC‐Malawi and Lowlaavar models, respectively, risked being dropped from the analysis because of the incomplete data in the required variables. Refer the Table S2. Owing to the substantial amount of missing data, we undertook multiple imputation by chained equations to address the challenge under the assumption of data missing at random, where data values are imputed using a set of univariate conditional imputation models^27^ and generate multiple “complete” datasets with different plausible values of the missing values. As recommended, we included all variables of interest from two models in the imputation model and selected other auxiliary variables in the database to preserve the relationship among variables,^28^, ^29^ giving a total of 53 variables in the imputation model. In the model, we specified different imputation options conditional on the variable type; for instance, ordered logistic regression option was applicable to ordinal categorical variables, the multinomial logistic regression for nominal multi‐level was applicable to categorical variables with more than 2 levels, and the binary logistic regression for dichotomous categorical variables. Based on the principle that the number of imputations must at least be equal to the proportion of incomplete data,^28^ we generated 70 multiply imputed datasets since 68% the records were incomplete. Graphical comparisons of the kernel density plots of the imputed versus observed values suggested plausible imputed values since the distributions of the values from the two datasets (imputed and original) appeared identical (Figure S2). Model recalibration strategy was then applied to each of the 70 imputed datasets and estimates pooled using Rubin's rules.^30^

### 2.8 Model recalibration strategy

Strategies for model recalibration include adding new predictors or updating the model's slope and intercept.^10^ Since the former strategy is akin to developing a new model which would require another external validation, we used the coefficient/intercept updating strategy to recalibrate the identified clinical prediction models to the local context. The original logistic regression model to be updated follows a standard format as shown in Equation 1 whereby the α denotes the model intercept and $\beta_{1}$ to $\beta_{p}$ denotes the vector of model coefficients (also called slope) for each prognostic factor$\;X_{1}$ to $X_{p}$ (also called covariate).

(1) $$\log\left[\frac{\mathrm{Pr}\left(\text{hospital mortality}\right)}{1-\mathrm{Pr}\left(\text{hospital mortality}\right)}\right]=\alpha +\beta_{1}X_{1}+\beta_{2}X_{2}+\cdots +\beta_{p}X_{p}$$

The right‐hand side of the Equation 1 constitutes the linear predictor ($LP_{\text{original}}$) of the original model which is a weighted sum of the prognostic factor$\;X_{1}$ to $X_{p}\;$in the model, weights being $\beta_{1}$ to $\beta_{p}$ which are the regression coefficients. This computation is done for each patient meeting the eligibility criteria of the two models (RISC‐Malawi and Lowlaavar) in the updating dataset. The resultant linear predictor is used by the recalibration strategies to adjust the model accordingly.^10^, ^31^ In this work, we explored two strategies namely, updating only model intercept (recalibration‐in‐the‐large), and updating of both the model intercept and slope (logistic calibration) as described below.

#### 2.8.1 Updating model intercept only

This method adjusts only the intercept of the original model such that the new intercept is equivalent to the average of the predicted in‐hospital probabilities in the updated dataset.^9^ This was achieved by fitting a univariable logistic regression model with an outcome of in‐hospital mortality, and the linear predictor was treated as an offset, thereby fixing the constant coefficient of the covariate at unity for each observation in the updating dataset. From this model, we obtained an intercept that was added to the linear predictor of the original model as a correction factor, but the regression coefficients ($\beta_{1}$ to $\beta_{p}$) of the original model remain unchanged as shown in Equation 2.

(2) $$\log\left[\frac{\mathrm{Pr}\left(\text{hospital mortality}\right)}{1-\mathrm{Pr}\left(\text{hospital mortality}\right)}\right]=\left(\alpha +\alpha_{\text{correction factor}}\right)+\beta_{1}X_{1}+\beta_{2}X_{2}+\cdots +\beta_{p}X_{p}$$

#### 2.8.2 Logistic calibration

This method updated both the model intercept and model slope simultaneously for each of the models we were updating. We fit a univariable logistic regression to each of the updating datasets, whereby in‐hospital mortality was treated as a dependent variable and the linear predictor as a covariate. The model yielded two correction factors that were used to adjust proportionally the original models' slope and intercept as shown in Equation 3.^31^ The advantage of the logistic calibration strategy is that the model intercept and slope of the original model are adjusted simultaneously, as opposed to the calibration‐in‐the‐large strategy, whose usefulness is only limited to updating the intercept of the original model based on the observed frequency of the outcome.

(3) $$\log\left[\frac{\mathrm{Pr}\left(\text{hospital mortality}\right)}{1-\mathrm{Pr}\left(\text{hospital mortality}\right)}\right]=\left(\alpha_{\text{correction factor}}\right)+\left(LP_{\text{original}}\times \beta_{\text{correction}\;\mathrm{fa}\text{ctor}}\right)$$

### 2.9 Assessing performance of the recalibrated prognostic models in the test dataset

For each model, we separately applied the two recalibration strategies (intercept only and logistic calibration) as described above. Using the recalibrated model in the model‐specific test dataset, we computed a linear predictor for each patient, which in turn was used to compute the patient's predicted risk of mortality via a logistic function. Model performance was determined using two metrics, namely discriminatory ability, and model calibration. Discriminatory ability was determined using the *c*‐statistic (value 0–1, discriminative if >0.7)^32^, ^33^ while the calibration was measured using the *calibration slope* that summarises agreement between predicted and observed risks where values equal to 1 are indicative of accurate predictions while those <1 and >1 suggestive of extreme and moderate risk predictions, respectively. *Calibration intercept* which indicates the extent that predictions are systematically too low or too high, with predicted risks being under‐estimated if >0 or over‐estimated if <0.^34^

We also used decision curve analysis to perform a head‐to‐head model comparison. To do this, we applied the eligibility criteria of the RISC‐Malawi and Lowlaavar models to the CIN population to find a common cohort for model comparison. The utility of decision curve analytics is to evaluate the clinical impact of implementing models in practice.^35^, ^36^ A model is of clinical utility if the net benefit of a model is greater than the scenario of “Treat all” (prioritise all patients) and “Treat none” (no patient is prioritised regardless of the risk of deterioration).

## 3 RESULTS

### 3.1 Patients' characteristics

The distribution of patient characteristics in the recalibrating and test datasets for RISC‐Malawi model was similar, although the test set had slightly higher mortality 1948 (9.6%) than the updating dataset 2458 (8.1%). This finding was not unexpected because in the cohort for model testing, cases of severe hypoxemia were 24%, which was almost twice that of model updating (13.4%), as shown in Table 1. However, we noted that cases of severe hypoxemia in the RISC‐Malawi original study were 12.7% which was comparable with that of the model updating dataset. For the Lowlaavar models, there were 10,782 patients meeting the eligibility criteria in 6 out of the 20 hospitals, with an overall in‐hospital mortality rate of 5.3%. A sub‐analysis to understand the distribution of mortality in the cohort revealed that mortality was higher (19.4%) among patients classified to have abnormal Blantyre Coma Score. In general, no appreciable differences were noted in the distributions of model predictors between updating and test datasets as shown in Table 2.

**TABLE 1:** Distribution of clinical characteristics of the cohort used to recalibrate and test RISC‐Malawi model

	Updating dataset (*n* = 30,343)	Test dataset (*n* = 20,326)	All patients (*N* = 50,669)
Mortality	2458 (8.1%)	1948 (9.6%)	4406 (8.7%)
Child‐sex (Female)	13,380 (44.1%)	8804 (43.3%)	22,184 (43.8%)
Age in months Median (Min, Max)	13.0 (2.00, 59.0)	13.0 (2.00, 59.0)	13.0 (2.00, 59.0)
Moderate hypoxemia^a^	1971 (6.5%)	1904 (9.4%)	3875 (7.6%)
Severe hypoxemia^b^	4071 (13.4%)	4878 (24.0%)	8949 (17.7%)
Moderately malnourished^c^	5245 (17.3%)	3454 (17.0%)	8699 (17.2%)
Severely malnourished^d^	1882 (6.2%)	1160 (5.7%)	3042 (6.0%)
Wheezing	3837 (12.6%)	2829 (13.9%)	6666 (13.2%)
Unconscious^e^	1774 (5.8%)	1447 (7.1%)	3221 (6.4%)

^a^ Defined as oxygen saturation 90%–92%.

^b^ Defined as oxygen saturation < 90%.

^c^ Defined as Mid‐Upper Arm Circumference (MUAC) of between 11.5 and 13.5 cm.

^d^ Defined as MUAC <11.5 cm.

^e^ Defined as either painful responsive or unresponsive in the disability scale of AVPU (Alert, Verbal, Painful responsive, unresponsive).

**TABLE 2:** Demographic and clinical characteristics of the cohort used to recalibrate and test Lowlaavar model

	Updating (*N* = 6175)	Test (*N* = 4607)	All patients (*N* = 10,782)
Mortality	327 (5.3%)	243 (5.3%)	570 (5.3%)
Child‐sex (Female)	2627 (42.5%)	1881 (40.8%)	4508 (41.8%)
Age in months Median (Min, Max)	24.0 (6.00, 60.0)	24.0 (6.00, 60.0)	24.0 (6.00, 60.0)
HIV diagnosis	52 (0.8%)	23 (0.5%)	75 (0.7%)
Abnormal Blantyre Coma Score	696 (11.3%)	400 (8.7%)	1096 (10.2%)
Weight for Age Z‐score Mean (SD)	−0.58(1.3)	−0.61(1.3)	−0.59 (1.33)
Mid‐upper Arm Circumference in centimetre (Min, Max)	14.2 (7.0, 21.0)	14.3 (8.6, 21.7)	14.3 (7.00, 21.7)

### 3.2 Predictive performance of the recalibrated models

The RISC‐Malawi model slope before recalibration was 1.04 (95% CI 1.00, 1.06) indicating regression coefficients were small (close to zero) and thus underestimating in‐hospital mortality predictions in the new patients. On the other hand, the calibration intercept was 0.81 (95% CI 0.77, 0.84), indicating that the predicted probabilities are systematically too low. The results of the intercept‐only method improved model intercept but suggested that recalibration of the slope was warranted, as provided in Supplementary File S1.

**FIGURE 2:**
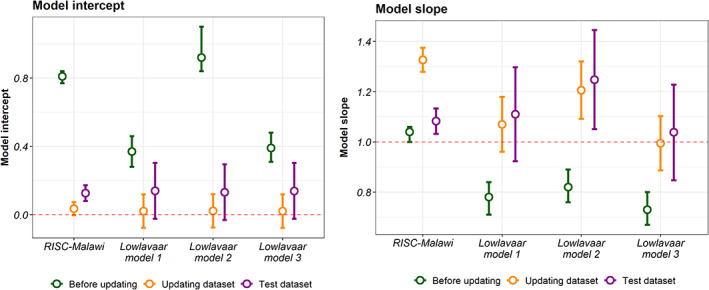
Calibration performance of the models in various datasets. The left panel shows calibration intercept while that on the right shows model slope. The coloured points and the 95% confidence intervals (shown as errors bars) shows the model calibration performances in the external validation, updating dataset (for model recalibration), and in the test dataset. The dotted line denotes the references of the model intercept (α = 0) and slope(β = 1) for a perfect calibrated model.

**FIGURE 3:**
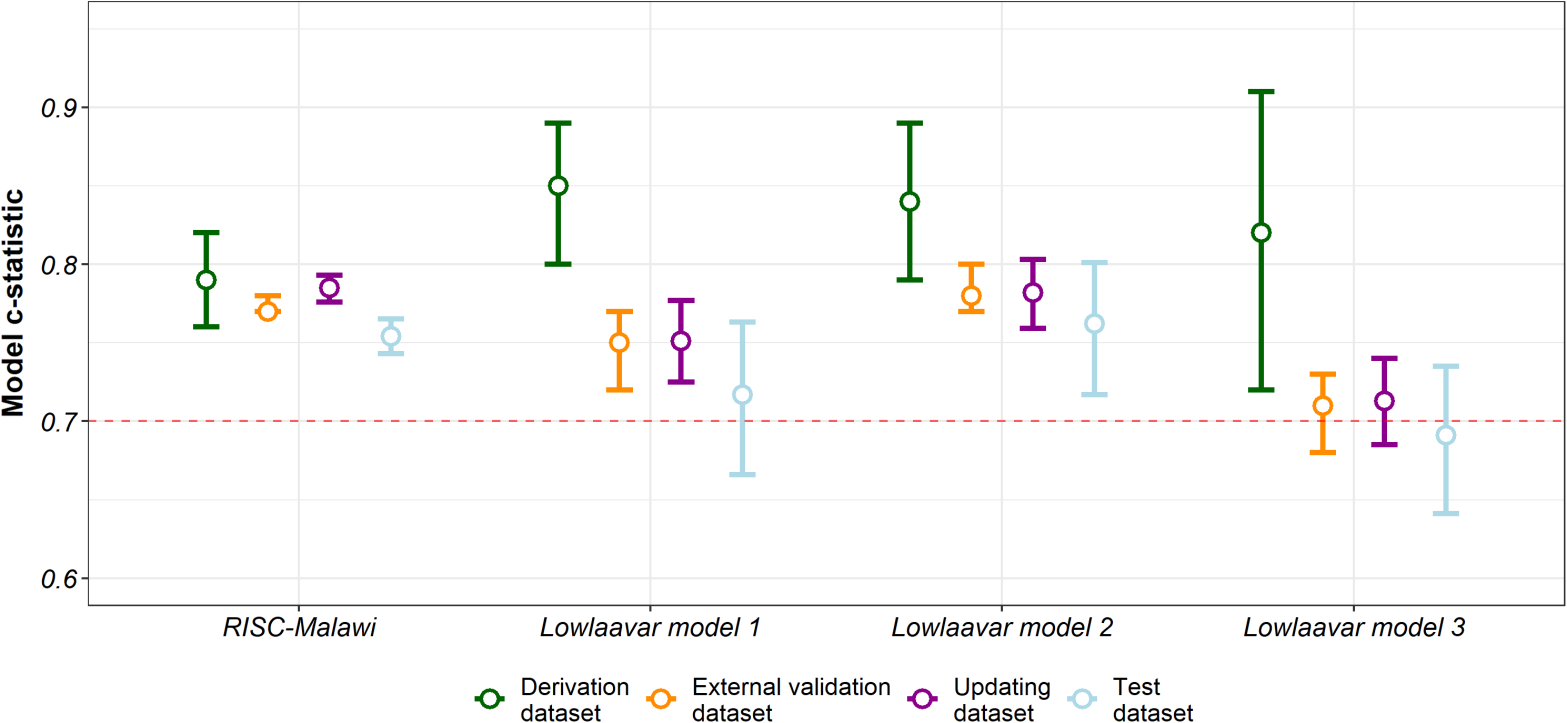
Discriminatory ability of the four models (RISC‐Malawi, and the 3 models by Lowlaavar et al) in various datasets. The coloured points and the 95% confidence intervals (shown as errors bars) shows the c‐statistics of the in the derivation dataset, external validation, updating (for model recalibration), and in the test dataset. The dotted line denotes a fair discriminatory ability of the model (c‐statistics of ≥0.7)

From the logistic calibration model of the RISC‐Malawi model, we obtained correction factors that were used in model adjustment. The adjusted model showed an improvement in the model intercept of 0.04 (95% CI −0.003, 0.07) compared with the original. However, upon assessing the same model in a test dataset, the model intercept deteriorated slightly to 0.13 (95% CI: 0.08–0.17), and the model slope also dropped to 1.08 (95% CI 1.03, 1.13) as shown in Figure 2. Compared with the derivation cohort, the discriminative ability of the RISC‐Malawi was not any different in the updating dataset with a c‐statistic of 0.78 (95% CI 0.78, 0.79) but was lower in the test set with a c‐statistic of 0.75 (95% CI 0.74, 0.76) as shown in Figure 3.

Calibration of the Lowlaavar models also suggested an improvement in the model intercept and slope (Figure 2). In general, all models exhibited improvements in calibration performance statistics relative to estimates before recalibration. However, none met the threshold of a perfectly calibrated model as judged by the slope and intercept estimates in the test dataset.

**FIGURE 4:**
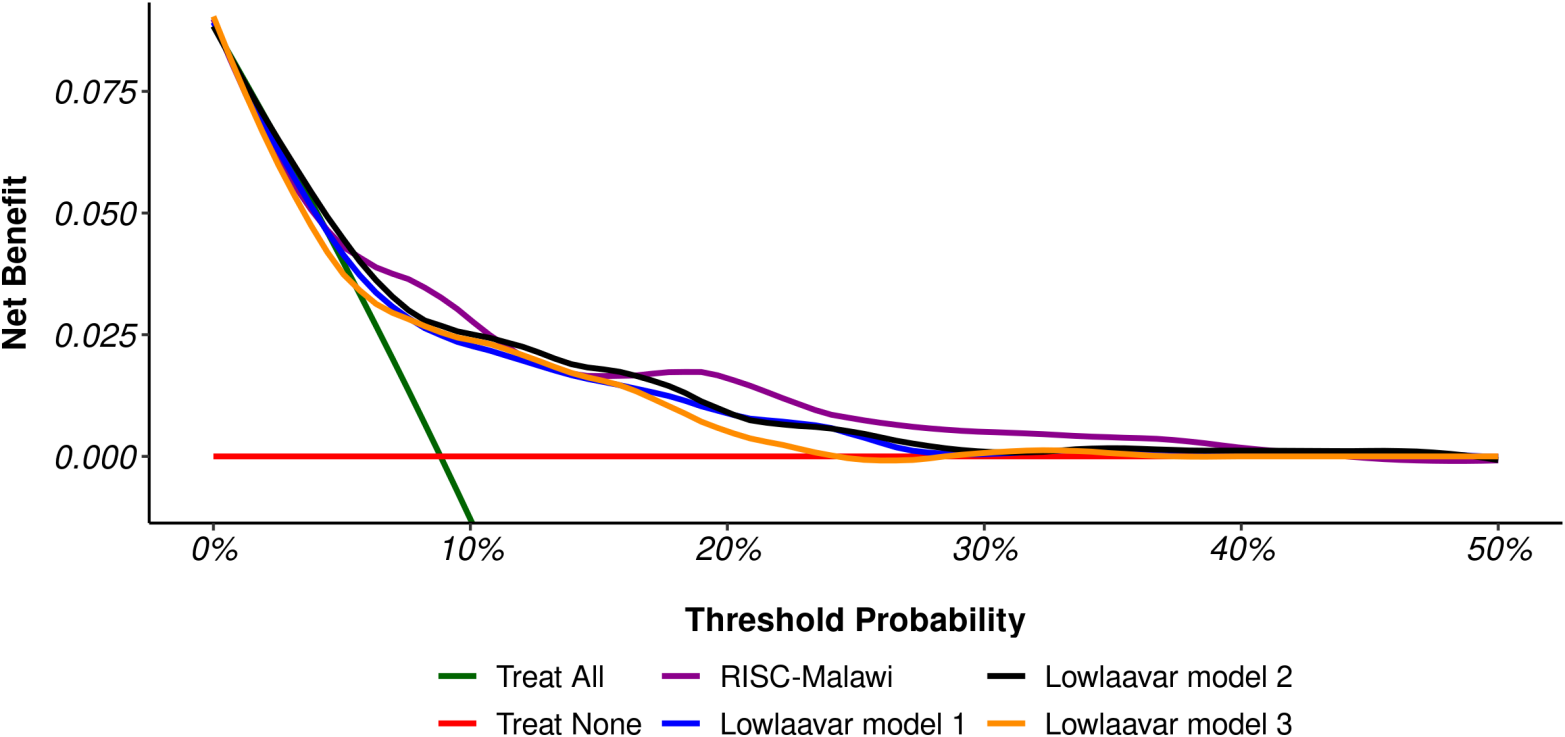
Decision curve analysis for the patients meeting the eligibility of all models. The “Treat All” line chart assumes all patients are at an increased risk of deterioration hence all should be prioritised for treatment, whereas the “Treat None” line chart assumes that no one is at the risk of deterioration hence none to be prioritised for treatment. The four coloured line charts show the net benefit of using models to identify patients at risk of deterioration.

Decision curve analysis was performed using 1120 patients who met the eligibility of all models. As shown in Figure 4, the curves diverge at the threshold probability of about 9% from the scenario of treating all patients. The analysis also shows that the RISC‐Malawi model's net benefit was slightly greater than all other models and the scenarios of “Treat All” as well as the scenario of “Treat None” for the predicted probability thresholds between 20% and 40%.

## 4 COMMENT

### 4.1 Principal findings

While the calibration of the models may have improved after recalibrating, they may not be clinically meaningful in practice as they are yet to meet the expected thresholds of a perfectly calibrated model (model intercept of 0, and a model slope of 1).

### 4.2 Strengths of the study

We explored avenues for improving the performance of the existing prognostic models based on the methodological strategies applied to large sample sizes powered enough to recalibrate and test models. In addition, the CIN data had both temporal and spatial richness with data collected from 20 county referral hospitals from 2014 to 2021.

### 4.3 Limitation of the data

The CIN datasets used to recalibrate and test the RISC‐Malawi model lacked the “unconsciousness” predictor requiring an auxiliary variable AVPU to gauge consciousness levels since it is used to assess patients' brain function.^21^ We therefore believe that our conclusions are still valid even though we used this proxy variable.

### 4.4 Interpretation

Since the objective of this work was not to refit models, the recalibration strategies employed here do not change the ranking of the patient's predicted risk of in‐hospital mortality, and as a result, do not affect the models' discriminatory ability. It is possible that a drop in AUC in the test dataset could be due to chance. Based on this understanding, Lowlaavar model 3's low AUC in the test dataset when compared with the derivation set underscores the need to validate published prognostic models across plausibly similar contexts to ascertain if the discriminatory ability is consistent in multiple validation datasets.

A miscalibrated prognostic model has been termed “clinically harmful” because it reduces the net benefit of its applicability in identifying risky patients for treatment.^37^ Therefore we conducted decision curve analytics, which suggested that no model yielded a substantial net benefit across the threshold probabilities suggestive of underestimating the mortality risk.

Suboptimal calibration performances of the updated models can be explained by predictor‐outcome associations having substantially different populations in derivation, updating, and the test dataset.^10^ For instance, when compared with the pneumonia case‐fatality in the derivation dataset of 3.2%, the dataset used to update and test the RISC‐Malawi model had a higher pneumonia case‐fatality of 8.1% and 9.6%, respectively, as shown in Figure 1. On the other hand, mortality in the dataset used to recalibrate and test Lowlaavar models was not any different from the derivation cohort.

While it is more common for researchers to develop new prognostic models and sometimes even without regard to methodological rigour,^3^, ^4^ there is growing interest among researchers to recalibrate existing models to align with local context and be applied in clinical practice if found to be suitable. However, in the literature of prognostic research, what constitutes acceptable differences between the expected calibration thresholds and the observed model calibration performances has not been established. Further, the number of external validations a prognostic model is expected to have been subjected to before model updating is justified is unknown. In addition, even if a predictive model would be subjected to repeated model recalibrations, it is likely that prediction performance will plateau where no further meaningful gain will be realised.^38^ Therefore, researchers might consider ensemble machine learning techniques such as stacking of point estimate or posterior predictive probabilities to combine the predictive abilities of various competing models to yield a meta‐model whose predictive performance would certainly be relatively better than that of a single model.^39^

## 5 CONCLUSIONS

Due to sampling variations, any model can perform slightly differently when applied to new patient samples. It is commonplace for researchers to develop new models, but this practise wastes information gleaned from previous prognostic modelling efforts and can lead to overfitting models lacking generalisability. We demonstrated that prognostic models can be updated using simple recalibration strategies and observed an improvement despite not meeting the expected calibration thresholds. This calls for a computational method to combine these models into one meta‐model to improve out‐of‐sample predictive performance.

## AUTHOR CONTRIBUTIONS

The roles of the contributors were as follows: RS, SA, and MO conceptualised the study. MO drafted the initial manuscript with SA, TT, JA, JN and RS, contributed to its development. All authors read and approved the final manuscript.

## Supporting information


supplementary file

## Data Availability

The data that support the findings of this study are available on request from the corresponding author. The data are not publicly available due to privacy or ethical restrictions.
