# Supplementary material for: Recalibrating prognostic models to improve predictions of in‐hospital child mortality in resource‐limited settings
Source: Paediatr Perinat Epidemiol. 2023 Feb 6;37(4):313–21. doi: 10.1111/ppe.12948 (PMC10946771; doi:10.1111/ppe.12948)
Supplement: Supplementary file 1 — supplementary file [file PPE-37-313-s001.docx]

**Supplimentary files**


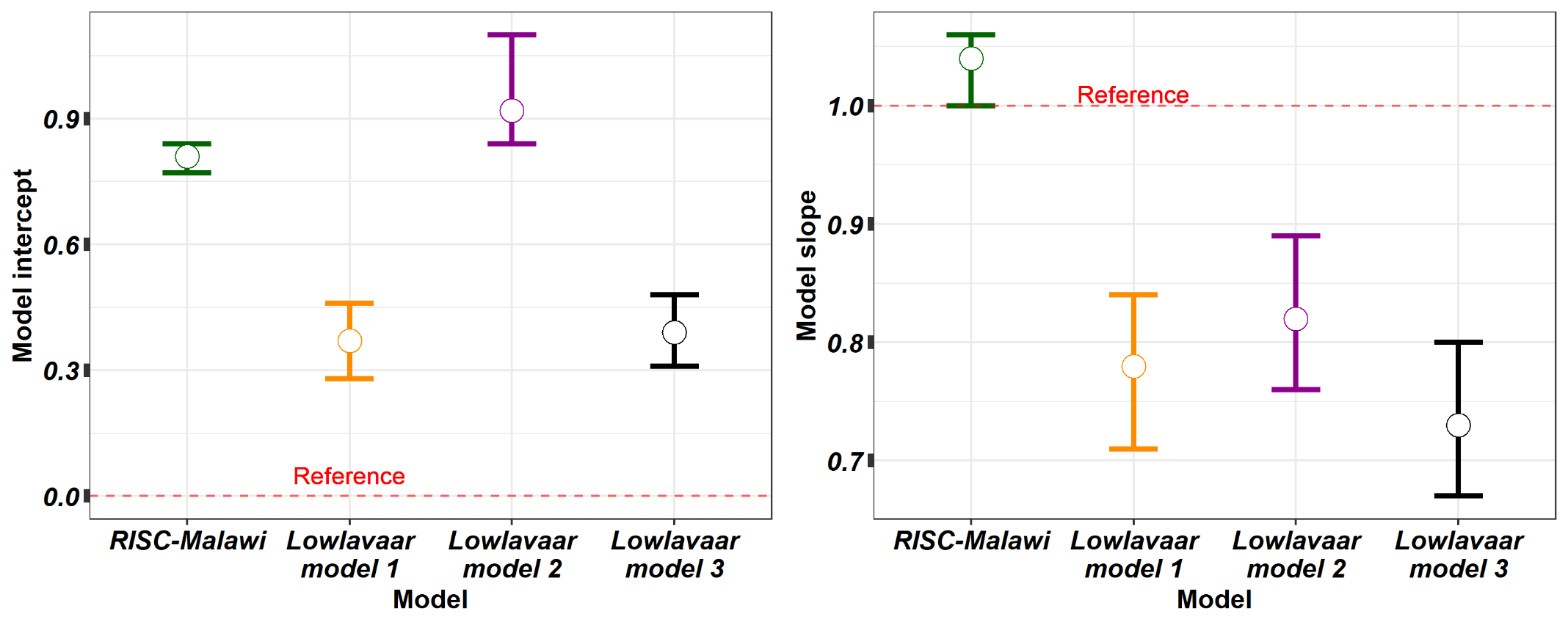


***eFigure 1: Model intercept and model slope of the four models suggesting that models were not well calibrated. These estimates were obtained from external validation study by Ogero et al ^1^***


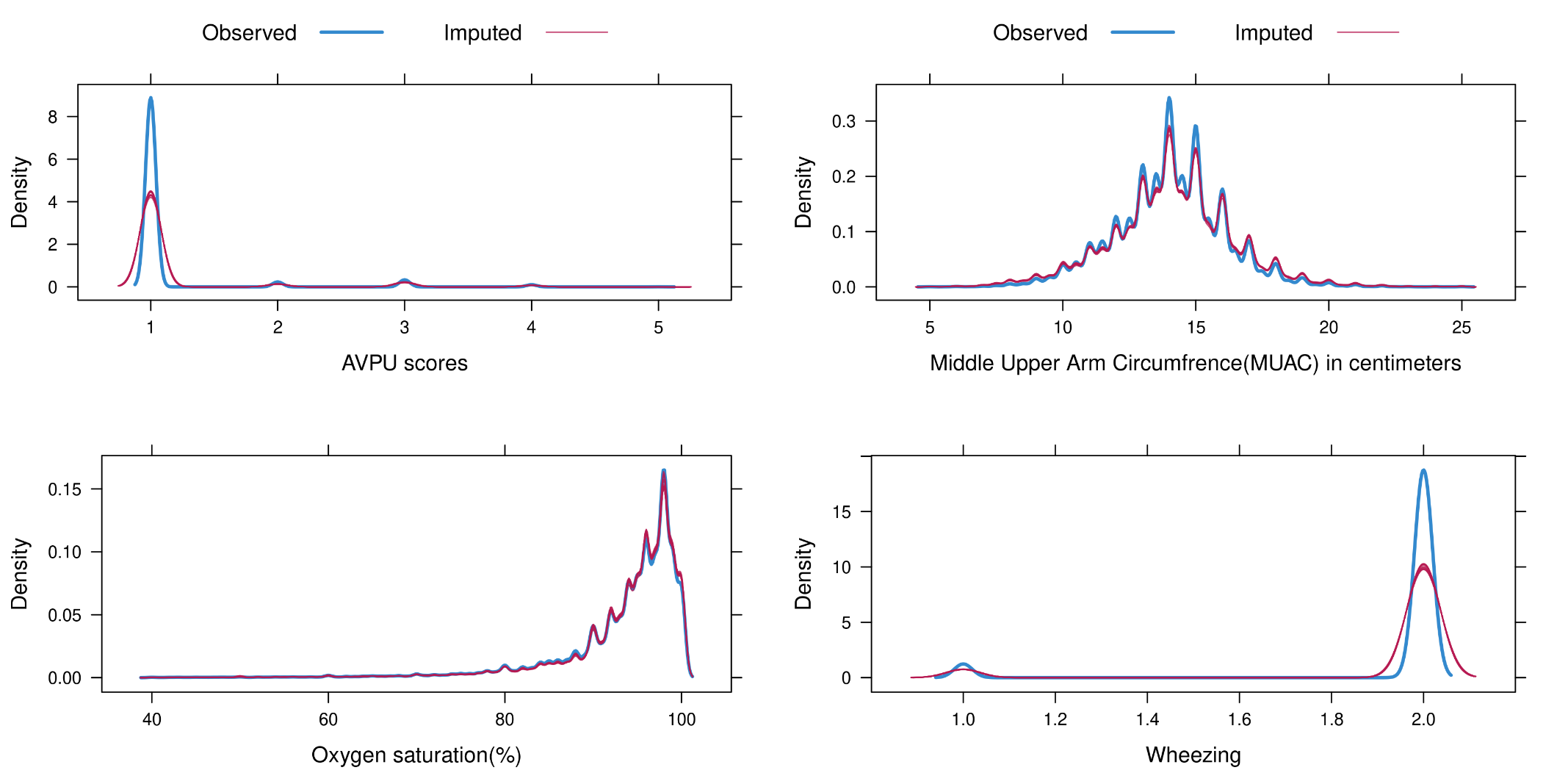
***eFigure 2: Kernel density plots of the observed (non-imputed) and imputed values of various variables. Visual inspection of the distributions of the observed and imputed values appears identical suggesting the imputation model generated plausible values to replace missing ones.***

**eTable 1: Minimum required sample sizes for recalibration of the models**

| **Model** | **c-statistic in the derivation cohort** | **Number of parameters in the original model** | **Outcome prevalence in the derivation cohort** | **Margin of error in estimation of intercept**  **(assumption)** | **Difference between apparent and adjusted R-squared** | **Minimum required sample size** |
| --- | --- | --- | --- | --- | --- | --- |
| RISC-Malawi model | 0.79 | 7 | 3.2% | 5% | 5% | 1619 |
| Lowlaavar model 1 | 0.85 | 3 | 5% | 5% | 5% | 285 |
| Lowlaavar model 2 | 0.84 | 3 | 5% | 5% | 5% | 307 |
| Lowlaavar model 3 | 0.82 | 2 | 5% | 5% | 5% | 239 |

**eTable 2: Extent of missing data in the model updating dataset**

| **Model** | **Predictor** | **Missing data** |
| --- | --- | --- |
| RISC-Malawi | Oxygen saturation | 20,947(41.3%) |
|  | Child sex | 332(0.66%) |
|  | Malnutrition using Middle Upper Arm Circumference (MUAC) | 25,232(49.8%) |
|  | Wheezing | 1,302(2.6%) |
|  | Unconsciousness using AVPU (Alert, Verbal, Painful responsive, unresponsive) scale | 1,533(3.0%) |
| Lowlaavar models | Blantyre coma score | 103(1.0%) |
|  | Weight for age z-score (WAZ) | 45(0.4%) |
|  | Mid-upper arm circumference (MUAC) | 531 (4.9%) |


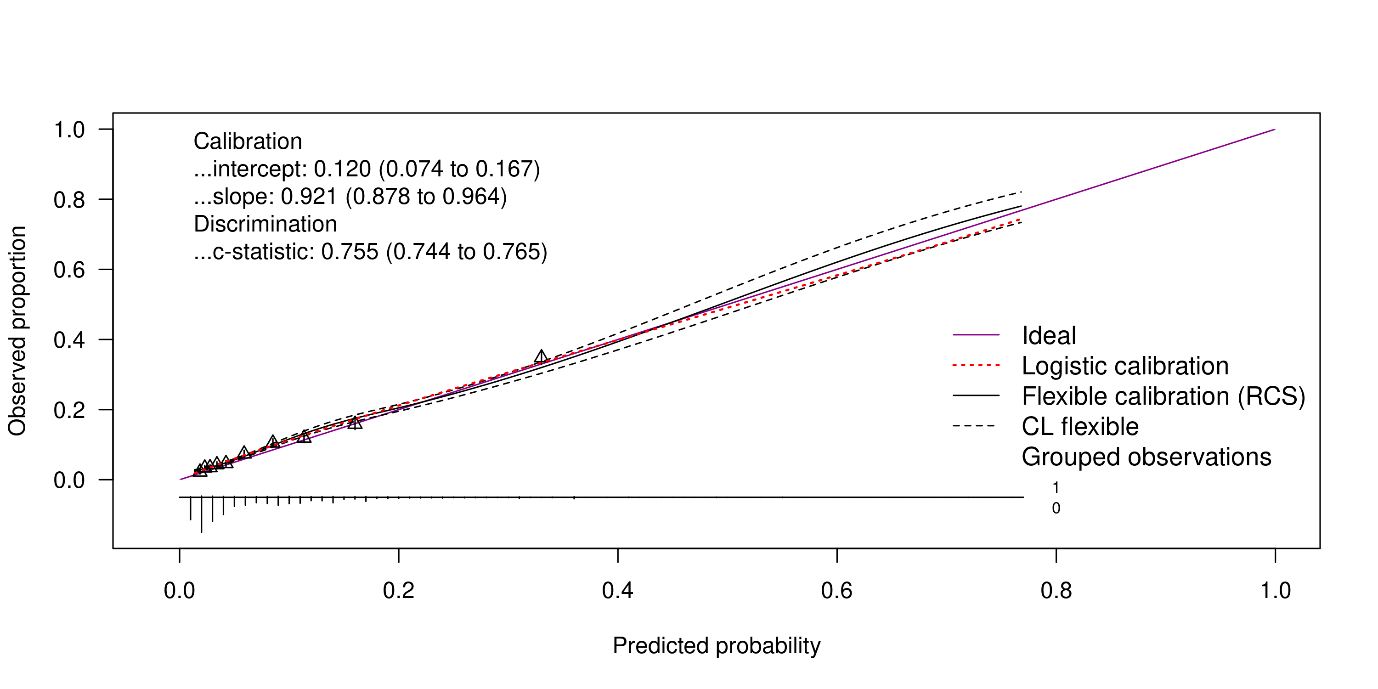


***eFigure 3*: Performance of the RISC-Malawi model in the test dataset after recalibrating using intercept only strategy. The figure shows calibration curves and other model performance metrics. Key: RCS denotes the Restricted Cubic Splines, and CL denotes the Confidence Limits (95%).**


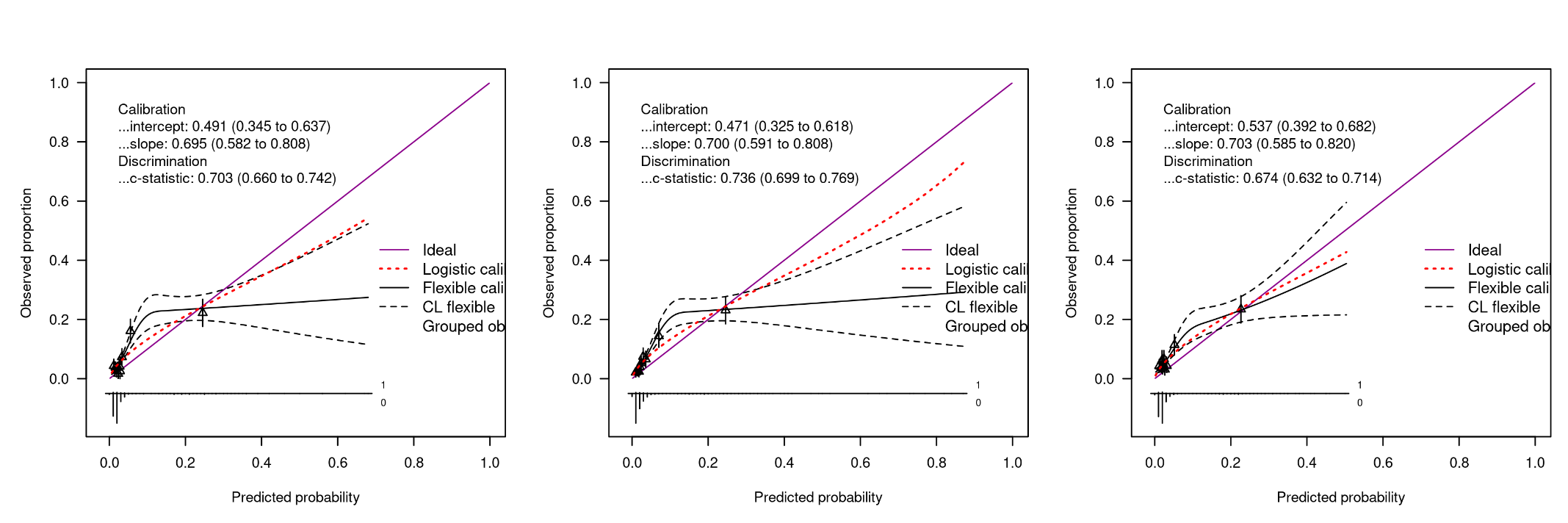
 ***eFigure 4*: shows calibration curves and performances of the Lowlaavar models in the test dataset after recalibrating using intercept only strategy. The panel on the left show performance of the Lowlaavar model 1, the one in the center shows Lowlaavar model 2, and that on the right is for Lowlaavar model 3. Key: RCS denotes the Restricted Cubic Splines, and CL denotes the Confidence Limits (95%).**

References

1. Ogero M, Sarguta R, Akech S. External validation of pediatric prognostic models predicting in-hospital child mortality in resource-limited settings. *BMJ Open* 2022
